# PET Imaging for Dynamically Monitoring Neuroinflammation in APP/PS1 Mouse Model Using [^18^F]DPA714

**DOI:** 10.3389/fnins.2020.00810

**Published:** 2020-09-29

**Authors:** Wei Hu, Donghui Pan, Yalin Wang, Weiqi Bao, Chuantao Zuo, Yihui Guan, Fengchun Hua, Min Yang, Jun Zhao

**Affiliations:** ^1^PET Center, Huashan Hospital, Fudan University, Shanghai, China; ^2^Department of Nuclear Medicine, Affiliated Wuxi People's Hospital, Nanjing Medical University, Wuxi, China; ^3^Key Laboratory of Nuclear Medicine, Jiangsu Key Laboratory of Molecular Nuclear Medicine, Jiangsu Institute of Nuclear Medicine, Ministry of Health, Wuxi, China; ^4^State Key Lab of Medical Neurobiology, Department of Integrative Medicine and Neurobiology, Institutes of Brain Science, Shanghai Medical College, Fudan University, Shanghai, China; ^5^Department of Nuclear Medicine, Shanghai East Hospital, Tongji University School of Medicine, Shanghai, China

**Keywords:** microglia, translocator protein (18 kDa), [^18^F]DPA714, PET, Alzheimer's diseases, APP/PS1

## Abstract

**Background:** In the pathogenesis of Alzheimer's disease (AD), microglia play an increasingly important role. Molecular imaging of neuroinflammatory targeting microglia activation and the high expression of 18-kDa translocator protein (TSPO) has become a hot topic of research in recent years. Dynamic monitoring neuroinflammation is crucial for discovering the best time point of anti-inflammatory therapy. Motivated by this, Positron emission tomography (PET) imaging in an APP/PS1 mouse model of AD, using ^18^F-labeled DPA-714 to monitor microglia activation and neuroinflammation, were performed in this paper.

**Methods:** We prepared [^18^F]DPA714 and tested the biological characteristics of the molecular probe in normal mice. To obtain a higher radiochemical yield, we improved the [^18^F]-fluorination conditions in the precursor dosage, reaction temperature, and synthesis time. We performed [^18^F]DPA714 PET scanning on APP/PS1 mice at 6–7, 9–10, 12–13, and 15–16 months of age, respectively. The same experiments were conducted in Wild-type (Wt) mice as a control. Referring to the [^18^F]DPA714 concentrated situation in the brain, we performed blocking experiments with PK11195 (1 mg/kg) in 12–13-months-old APP/PS1 mice to confirm the specificity of [^18^F]DPA714 for TSPO in the APP/PS1 mice. Reconstructed brain PET images, fused with the Magnetic Resonance Imaging (MRI) template atlas, and the volumes of interests (VOIs) of the hippocampus and cortex were determined. The distribution of [^18^F]DPA714 in the brain tissues of 15–16-months-old APP/PS1 and Wt mice were studied by immunofluorescence staining.

**Results:** Through the reaction of ^18^F, with 2 mg precursor dissolved in 300 ul acetonitrile at 105°C for 10 min, we obtained the optimal radiochemical yield of 42.3 ± 5.1% (non-decay correction). Quantitative analysis of brain PET images showed that the [^18^F]DPA714 uptake in the cortex and hippocampus of 12–13-months-old APP/PS1 mice was higher than that of the control mice of the same age (cortex/muscle: 2.77 ± 0.13 vs. 1.93 ± 0.32, *p* = 0.0014; hippocampus/muscle: 3.33 ± 0.10 vs. 2.10 ± 0.35, *p* = 0.0008). The same significant difference was found between 15- and 16-months-old APP/PS1 mice (cortex/muscle: 2.64 ± 0.14 vs. 1.86 ± 0.52, p=0.0159; hippocampus/muscle: 2.89 ± 0.53 vs. 1.77 ± 0.48, *p* = 0.0050). Immunofluorescence staining showed that the activation of microglia and the level of TSPO expression in the cortex and hippocampus of APP/PS1 mice were significantly higher than Wt mice.

**Conclusion:** [^18^F]DPA714, a molecular probe for targeting TSPO, showed great potential in monitoring microglia activation and neuroinflammation, which can be helpful in discovering the best time point for anti-inflammatory therapy in AD.

## 1. Background

Alzheimer's disease (AD) is the chronic inflammation of the central nervous system (CNS), including focal brain injury and a lot of beta-amyloid protein (A beta, Aβ) deposition (Golde, 2016). In recent years, it has been found that microglia play a major role in the pathogenesis of AD, which is also currently a hot topic in the study of neurodegenerative diseases. Microglia activated by Aβ depositions is one of the main mechanisms of neuroinflammation. Activated microglia produce a large number of precursors of inflammatory factors, resulting in the occurrence of neurodegenerative diseases (Politis et al., 2012; Li et al., 2014). The translocator protein (TSPO, 18 kDa), also known as the peripheral benzodiazepine receptor (PBR), is mainly localized in the outer mitochondrial membrane of steroid-synthesizing cells of the peripheral and central nervous system. Normally, the central nervous system is restricted to glial cells (astrocytes and microglia) which are expressed less in the CNS. The expression of TSPO in microglia is significantly increased in response to injury or inflammatory of the brain disease, so it can be used as an important neuroinflammation marker (Chen and Guilarte, 2007; Rupprecht et al., 2012).

Although a lot of research has been done, the role of activated microglia in the dynamic stages of neuroinflammation is still unclear. Positron emission tomography (PET) tracers targeting TSPO has been widely used to detect the activation of microglia in both preclinical and clinical studies (James and Gambhir, 2012). [^11^C]PK11195 is the first tracer used in neuroinflammatory PET imaging studies. Although it has been studied for more than 20 years, [^11^C]PK11195 still has many limitations: the specificity of [^11^C]PK11195 binding to TSPO is low, which affects the image quality and quantitative analysis of microglia activation; furthermore, carbon-11 has a short half-life (20.38 min) and requires field cyclotron for nuclide preparation, which limits its wide clinical application (Chauveau et al., 2008). In the past decade, many researchers have improved the properties of TSPO tracers and developed new specific radio-ligands, including [^18^F]DPA714 (Vivash et al., 2016). [^18^F]DPA714 is a pyrazolopyrimidine TSPO ligand with high affinity, which has a good stability in plasma and brain tissue and has been used in animal and human PET imaging studies (James et al., 2008; Arlicot et al., 2012; Peyronneau et al., 2012). Compared with [^11^C]PK11195, [^18^F]DPA714 has a significant improvement in the affinity and decrease of non-specific binding to TSPO (Chauveau et al., 2009). The purpose of this study is to evaluate the role of [^18^F]DPA714 PET imaging in monitoring the TSPO levels in the mouse model of APP/PS1 mice at different months, which can help us to better understand the action of microglia in the pathogenesis of AD neuroinflammation. To the best of our knowledge (Takkinen et al., 2016) is the only research that shares a similar aim to our paper. It is a longitudinal PET study which demonstrated decreased energy metabolism and increased inflammation in the brains of APP/PS1-21 mice compared to WT mice. Our work, however, differs in terms of experimental method and design: (1) The radioactive labeling condition of [^18^F]DPA714 is optimized in our work, which increases the radiochemical yield with fewer precursors and a shorter synthesis time; (2) In this work, PET dynamic imaging on mice were performed directly after projection, which was crucial in determining the best time point for PET imaging after injection. (3) A blocking experiment using PK11195 demonstrated that [^18^F]DPA714 had improved specificity for TSPO.

## 2. Method

### 2.1. General Materials

All chemicals used are of analytical grade and were purchased from Aldrich and Sigma. [^18^F]-F-radionuclide was obtained from Jiangsu Institute of Nuclear Medicine. We conducted mouse PET imaging on an Inveon micro-PET scanner (Siemens Medical Solutions,Germany). We received the precursor DPA714 from Professor Xiaoyuan Chen at the National Institutes of Health (NIH).

### 2.2. Animals

B6.Cg-Tg (APPswe, PSEN1dE9)85Dbo/Mmjax (APP/PS1) and Wt mice were purchased from Shanghai Model Organisms Center, Inc. and bred by the Jiangsu Institute of Nuclear Medicine. The provenances were obtained from the Jackson Laboratory. According to the Jackson Laboratory website's description, APP/PS1 mice could express chimeric mouse/human amyloid precursor protein (Mo/Hu APP695swe) and mutant human presenilin1 (PS1-dE9), both directed to the neurons of central nervous system, which is associated with early-onset Alzheimer's disease. The APP/PS1 mice produce β-amyloid deposits in the brain by 6–7 months of age and have elevated beta-amyloid levels between 6 and 12 months of age (Jankowsky and L., 2003; Garcia-Alloza et al., 2007; Xiong et al., 2011). APP/PS1 and Wt mice were identified by PCR assays of tail DNA. They were housed separately in an enriched environment with a 12-h light/12-h dark cycle, constant temperature (23 ± 1°C), and humidity (55 ± 5%) with available food and water.

### 2.3. Synthesis of [^18^F]DPA714

Based on previous reports (James et al., 2008; Arlicot et al., 2012; Wang et al., 2014), we have improved the labeling method. ^18^F-Fluoride was separated from ^18^O-H_2_O by QMA-Light Sep-Pak anion-exchange resin (Waters) preconditioned with 10 ml of 0.5 M KHCO_3_ and 15ml deionized water. Then ^18^F-Fluoride was eluted from Waters QMA anion exchange cartridge and transferred to the reaction vessel (Thermo Fisher Scientific, 2 ml) with eluent solution containing K_2_CO_3_ (3 mg in 10 ul of pure water), acetonitrile (90 ul), and 15 mg of Kryptofix [2.2.2] (Sigma, America). The solution was dried completely by azeotropic distillation with acetonitrile (3 × 1.0ml) (Acros, Belgium) under the mild flow of nitrogen at 110°C. Then, DPA-714 (2 mg) dissolved in 500 ul (4 mg/ml) or 300 ul (6.7 mg/ml) acetonitrile (Acros, Belgium) was added to the dry ^18^F-labeled K-F-Kryptofix-222 complex. The reaction mixture was heated at 95 or 105°C for 5 or 10 min and developed a brown color. After reaction, we diluted the mixture with sterile water and then separated it through a tC18 cartridge (Waters Sep-Pak Accell Light tC18 cartridge washed with 10 ml of ethanol and 15 ml deionized water before use). The products gathered by the tC18 cartridge were washed with deionized water (20 ml) and then eluted with acetonitrile (200 ul). The crude mixtures were injected into a Waters X-Terra C_18_10-mm (250 × 10 mm, Phenomenex, America) semi-preparative reversed-phase high-performance liquid chromatography (HPLC) column with a mobile phase of CH_3_CN, deionized water and triethylamine (50:50:0.1, v/v/v) at a flow rate of 3.0 mL/min. The purified products were eluted with 0.3 ml ethanol from tC18 cartridge and diluted with 0.9% saline to make [^18^F]DPA714 could be used for intravenous injection. The final solution with known volume and radioactivity were injected into a Waters X-Terra tC18 (250 × 4.6 mm, Phenomenex, America) analytic reversed-phase HPLC column to determine its radio-chemical purity. [^18^F]DPA714 was eluted with a mobile phase of CH_3_CN, deionized water and triethylamine (40:60:0.1, v/v/v) at a flow rate of 1.0 mL/min.

### 2.4. PET Scanning and Image Fusion

The study was performed using four female and four male APP/PS1 mice (APP/PS1, *n* = 8), and four female and four male Wt mice as the control group (Wt, *n* = 8). These 16 mice were scanned at ages of 6–7, 9–10, 12–13, and 15–16 months continuously. PET acquisitions were performed on a micro-PET (Inveon,Siemens) which had an effective axial/transaxial field of view (FOV) of 12.7/10 cm. Prior to the scans, mice were anesthetized using isoflurane (2.0–3.0% for induction and 1.5–2.5% for maintenance). They were placed into the scanner so that their brains were located in the center of the FOV. Dynamic scans (60 min) for APP/PS1 and Wt mice were performed after intravenous injection of [^18^F]DPA714(3.7–5.5 MBq, 0.2 ml). We conducted blocking studies, which pre-treated 12–13-months-old APP/PS1 mice with PK11195 (1 mg/kg; Sigma Aldrich) 10 min before radioligand administration. PK11195 is a TSPO antagonist. Pre-treatment with PK11195 resulted in saturation of the TSPO receptors, blocking them for [^18^F]DPA714 occupancy (Keller et al., 2018). All list-mode data were reconstructed using the OSEM 2D algorithm (frames, 4 × 30, 5 × 150, 6 × 450 s) with no attenuation correction. In the image analysis software PMOD (3.7, PMOD Technologies, Zurich, Switzerland; www.pmod.com), the reconstructed PET measurements were manually aligned with a mice brain MRI template atlas. We selected the hippocampus and cortex as the VOIs and calculated their percentages of injected dose per gram (%ID/g). Time-activity curves (TACs) were calculated as a mean of the VOIs and normalized for the %ID/g. To calculate the signal-to-background ratios, we divided the uptake of VOIs by the intake of reference region (muscle). For muscle VOIs, two regions were drawn per arm of each mouse (i.e., four muscle regions per mouse) using the PET image, and then the average %ID/g from all four muscle VOIs for each mouse was calculated to provide the overall muscle %ID/g (James et al., 2015).

### 2.5. Immunofluorescence Staining

The 15–16-months-old Wt and APP/PS1 mice were perfused immediately after PET imaging to take whole brain tissue samples. Through CM-1900 cryostat (Leica, Germany), we obtained mice brain cryosections with a thickness of 30 um. To quantify Iba-1 and TSPO staining, three sections per mouse in the 15–16-months-old group (*n* = 8 Wt and *n* = 8 APP/PS1) were analyzed. All the sections taken from the cryoprotectant were gently washed with 0.01 M Phosphate Buffered Saline (PBS) for 10 min, and then we blocked the non-specific binding sites with PBS containing 1% bovine serum albumin for 1 h. The treated slices needed to be incubated overnight with the primary antibodies at 4°C and then with the secondary antibodies at room temperature for 1 h. For different staining targets, it is critical to choose the specific antibodies and appropriate concentrations.

The antibodies and concentrations we used are as follows: goat anti-mouse Iba-1 (a microglial marker) antibody (1:500, ab5076, Abcam), rabbit anti-mouse PBR (TSPO) antibody (1:200, sc-20120, Santa Cruz); FITC-conjugated donkey anti-goat antibody (1:1,000, 1608643, Life technologies), FITC-conjugated donkey anti-rabbit antibody (1:1,000, 1674651, Life technologies). All tissue slices were mounted with medium containing 4,6-diamidino-2-phenylindole (DAPI, G3016-TI96, Southern Biotech) and then observed using a confocal laser scanning microscope (Leica, Germany).

One immunofluorescence image corresponds to each section of one mouse, and we have 8 Wt mice and 8 APP/PS1 mice with three sections per mouse. Therefore, we obtained 48 immunofluorescence figures of Iba-1 and TSPO after staining. These figures were imported into Image J software (1.42q) and results were analyzed and compared. The software first calculated mean gray value (mean) of each figure, which represented the figure fluorescent intensity and can be obtained as following equation: Mean Gray Value = Integrated Density/Area. Based on the mean gray values of both Iba-1 and TSPO, we drew the relative fluorescence intensity chart to compare them visually. Furthermore, the *T*-test was conducted to test differences for Wt and APP/PS1 mice. Figures of Dapi, Iba-1, and TSPO in the same field of vision were also merged by Image J software to observe the overlapping situation of three staining target areas.

### 2.6. Statistical Analysis

We presented all the data in the form of means ± standard deviation (mean ± SD). Statistical analysis was conducted using Graph-Pad Prism (version 5.1). Difference < 0.05 was considered to be statistically significant.

## 3. Results

### 3.1. Radiochemistry

**Figure 1:**
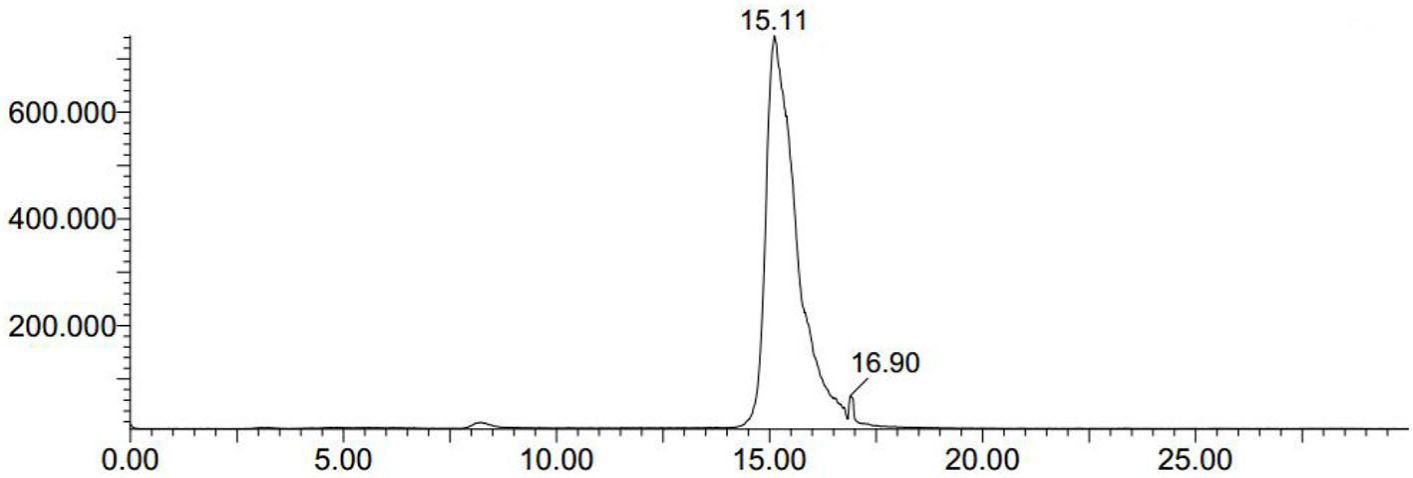
The crude mixtures of [^18^F]DPA714 was extracted using semi-preparative reversed-phase HPLC. The retention time (tR) of [^18^F]DPA714 was 15.11 min with a mobile phase of CH_3_CN, deionized water and triethylamine (50:50:0.1, v/v/v), liquid pump flow rate 3.0 mL/min.

**Figure 2:**
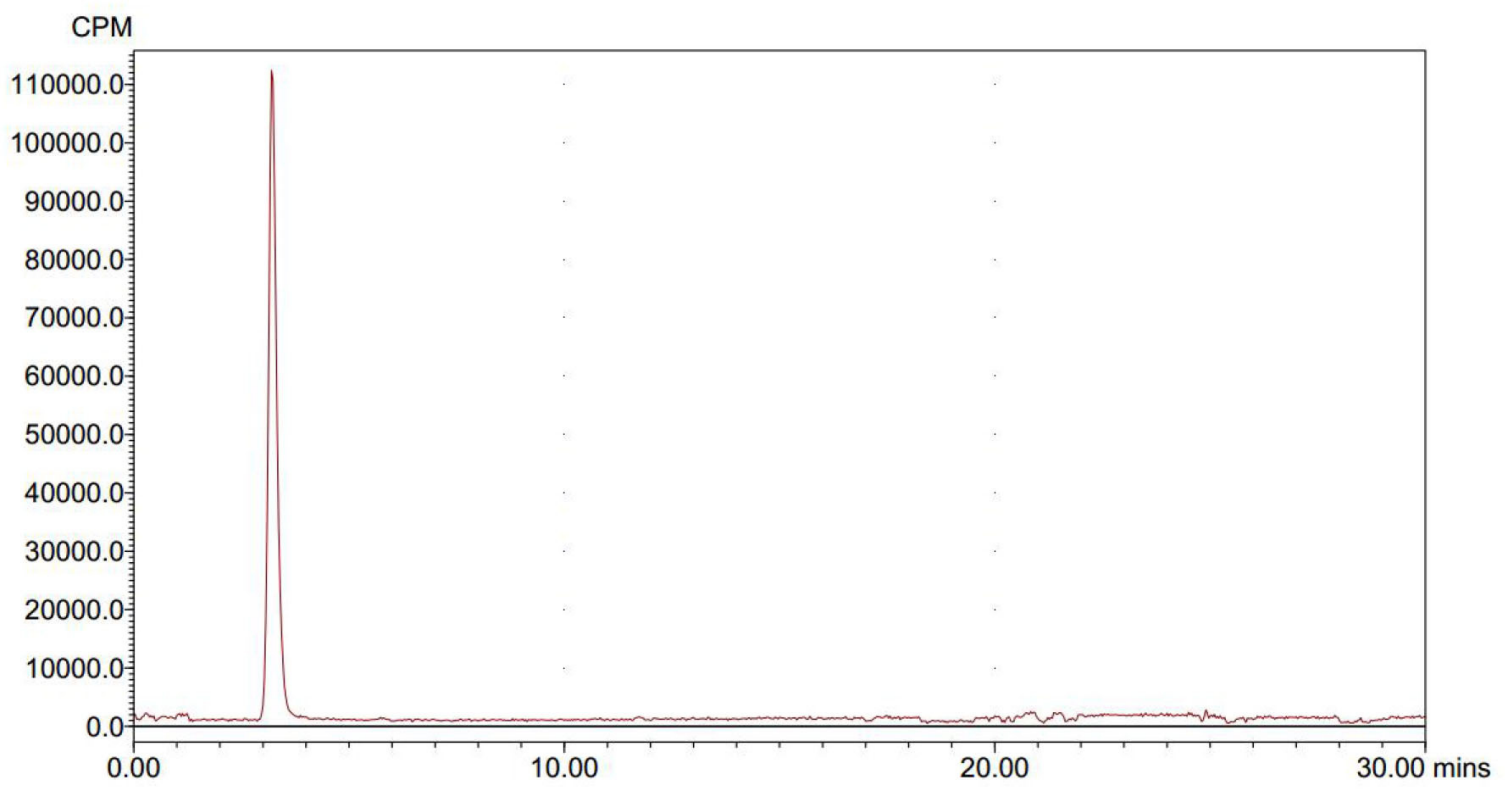
The final solution of [^18^F]DPA714 was injected into a analytic reversed-phase HPLC to determine its radio-chemical purity, the retention time (tR) of [^18^F]DPA714 was 3.5 min with a mobile phase of CH_3_CN, deionized water and triethylamine (40:60:0.1, v/v/v) setting the flow rate at 1.0 mL/min.

We conducted radiolabeling of DPA-714 and ^18^F by nucleophilic aliphatic substitution and selected the optimal concentration, temperature and reaction time of the precursor. Through the reaction of ^18^F with 2 mg precursor dissolved in 300 ul acetonitrile at 105°C for 10 min, we obtained the optimal radiochemical yield of 42.3 ± 5.1% (non-decay correction) (Table 1). Reaction time is another important condition affecting the radiochemical yield (Table 2). With the increase of the reaction time, the rate of radiochemical yield also increased. Total radio-synthesis time for the preparation of [^18^F]DPA714 was 30–35 min. The retention time (tR) of [^18^F]DPA714 in the semi-preparative reversed-phase HPLC system was 15.11 min (Figure 1) and the radiochemical purity of [^18^F]DPA714 is 99.9% (Figure 2).

**Table 1:** Result of the different temperature and concentration of precursor of the [^18^F]-fluorination yield of [^18^F]DPA714 with 10 min reaction time (non-decay-corrected; *n* = 4).

**Temperature (^**°**^C)**	**Concentration of precursor (mg/ml)**	
	**4**	**6.7**
95	16.3 ± 5.0%	31.6 ± 3.7%
105	25.4 ± 3.8%	42.3 ± 5.1%

**Table 2:** Result of the different reaction time on the [^18^F]-fluorination yield of [^18^F]DPA714 at 105°C reaction temperature (non-decay-corrected; *n* = 4).

**Reaction time (min)**	**Concentration of precursor (mg/ml)**	
	**4**	**6.7**
5	9.0 ± 0.6%	21.5 ± 1.0%
10	25.4 ± 3.8%	42.3 ± 5.1%

### 3.2. Mice PET

**Figure 3:**
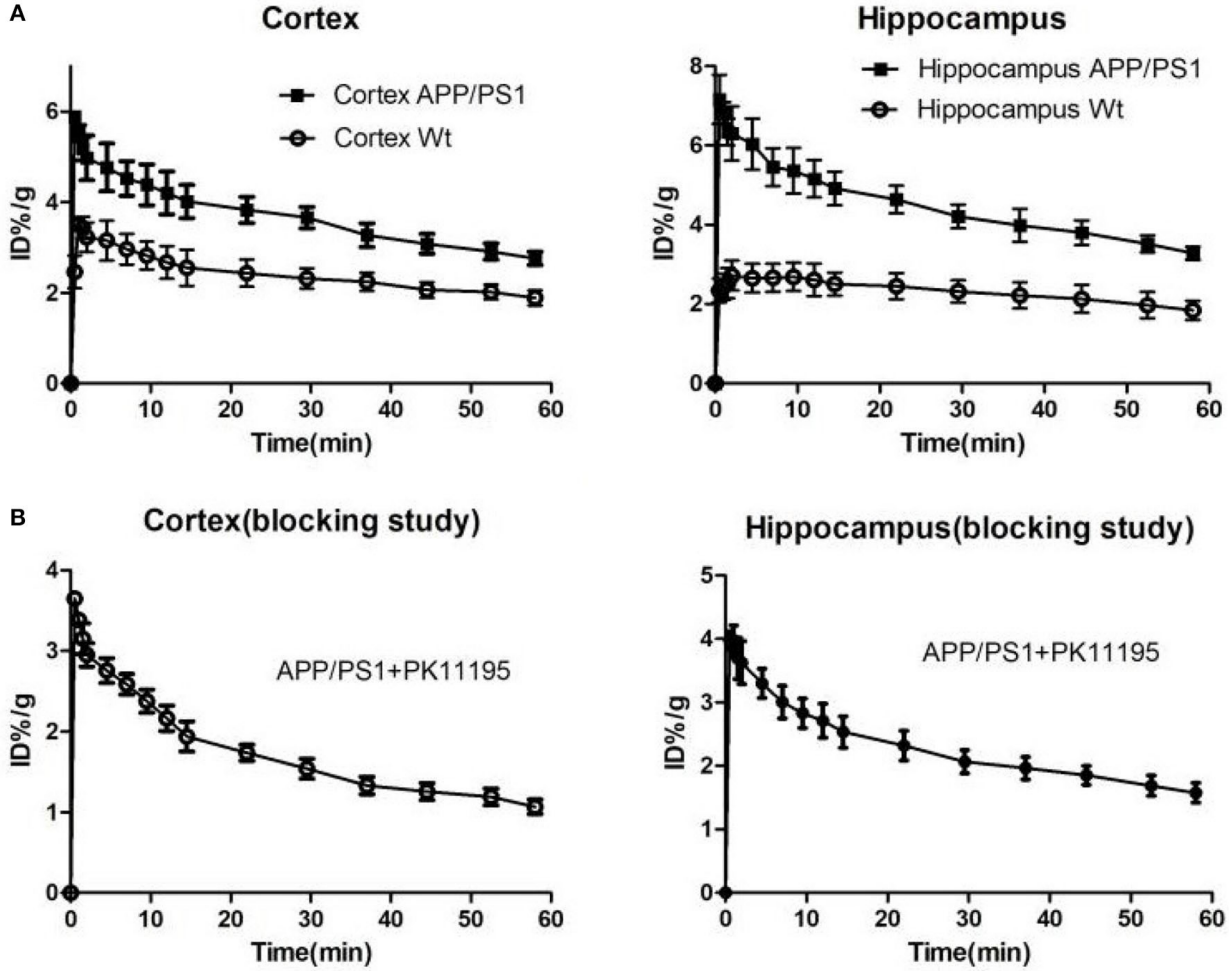
**(A)** The time-activity curves (TACs) of [^18^F]DPA714 accumulation in cortex and hippocampus of 12–13-months-old APP/PS1 (*n* = 8) and Wt (*n* = 8) mice using dynamic scan (60 min). **(B)** In blocking studies, the time-activity curves (TACs) of [^18^F]DPA714 accumulation in cortex and hippocampus pre-treated 12–13-months-old APP/PS1 mice (*n* = 8) with PK11195 10 min before radioligand administration.

We used [^18^F]DPA714 micro-PET imaging to monitor the TSPO levels of APP/PS1 mice in different ages and found that the microglia of different aged mice displayed different degrees of activation. Quantitative analysis of [^18^F]DPA714 PET images revealed that there was no significant difference in cortical and hippocampal signals between APP/PS1 and Wt mice aged 6–7 and 9–10 months. Conversely, APP/PS1 and Wt mice aged 12–13 and 15–16 months showed a significant difference, known to display substantial microglia activation. We performed [^18^F]DPA714 dynamic PET imaging for APP/PS1 and Wt mice. The time-activity curves (TACs) of dynamic imaging (Figure 3A) showed that [^18^F]DPA714 uptake in the cortex and hippocampus of 12–13-months-old APP/PS1 mice was significantly higher than that of Wt mice at 40–50 min post-injection (cortex: 3.08 ± 0.17 vs. 2.06 ± 0.22 %ID/g, ^*^P < 0.05; hippocampus: 3.80 ± 0.44 vs. 2.13 ± 0.49 %ID/g, ^**^P < 0.005). In the blocking experiments, we used PK11195 (1 mg/kg) a known TSPO ligand to block the signals in the cortex and hippocampus of 12–13-months-old APP/PS1 mice before tracer injection (Figure 3B).

**Figure 4:**
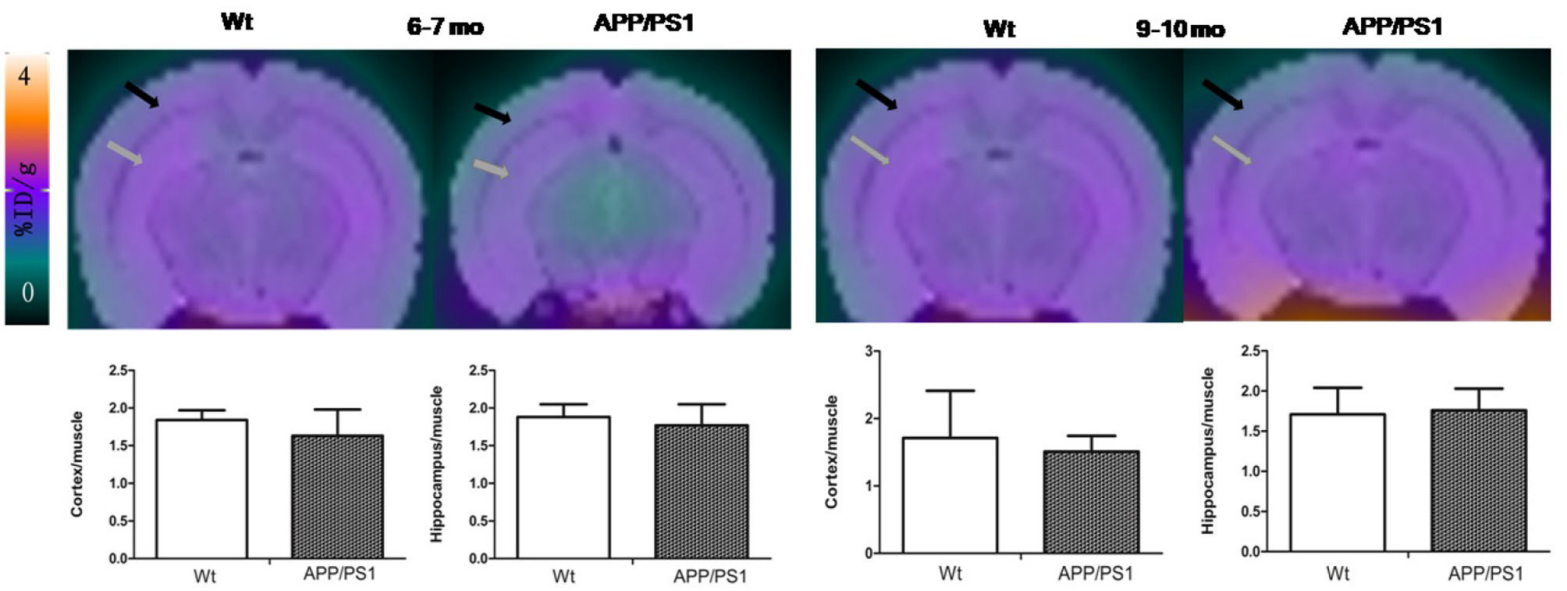
The PET images were aligned with mice brain MRI template atlas. We selected hippocampus and cortex as the VOIs and calculated their percentages of injected dose per gram (%ID/g). PET/MRI template atlas fusion images and graphs representing [^18^F]DPA714 signals in APP/PS1 vs. Wt mice 6–7 (*n* = 8) and 9–10 (*n* = 8) months of age. Black and gray arrows point to cortex and hippocampus, respectively.

**Figure 5:**
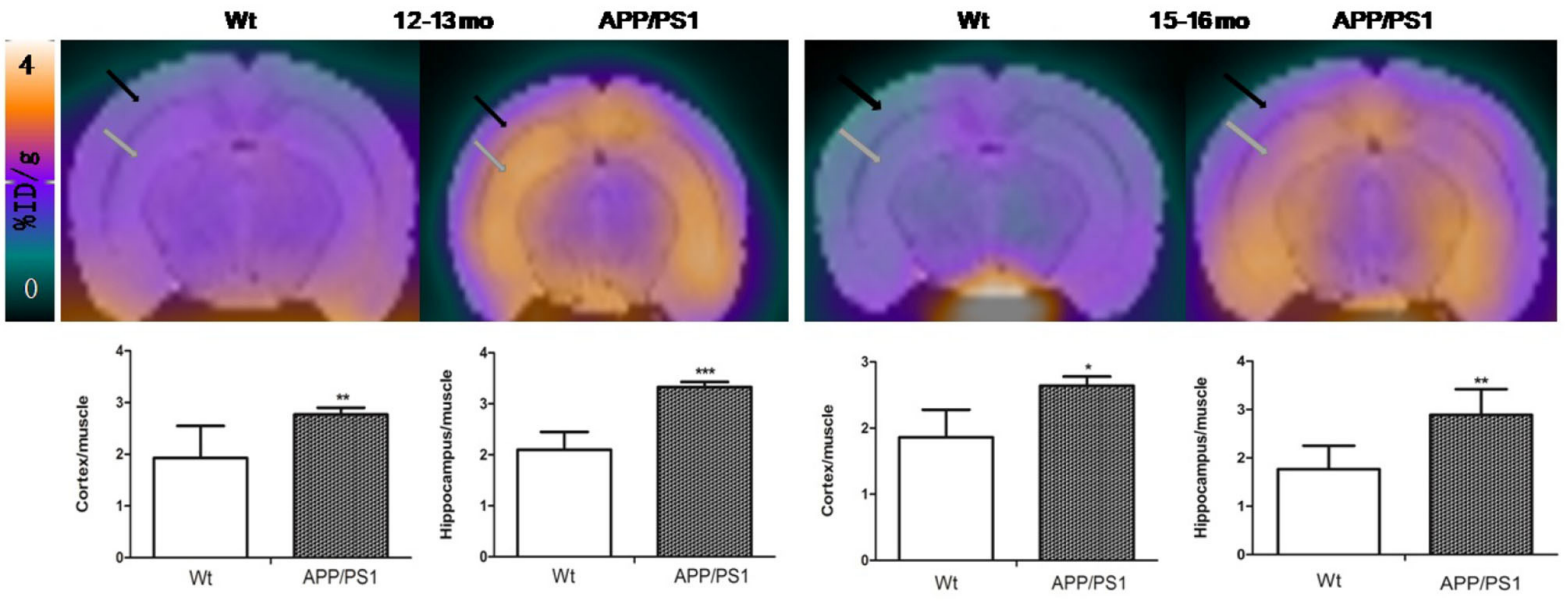
The PET images were aligned with the mouse brain MRI template atlas. We selected the hippocampus and cortex as the VOIs and calculated their percentages of injected dose per gram (%ID/g). PET/MRI template atlas fusion images and graphs representing [^18^F]DPA714 signals in APP/PS1 vs. Wt mice 12–13 and 15–16 months of age. Black and gray arrows point to cortex and hippocampus, respectively (^*^p < 0.05, ^**^p < 0.01, ^***^p < 0.001).

**Figure 6:**
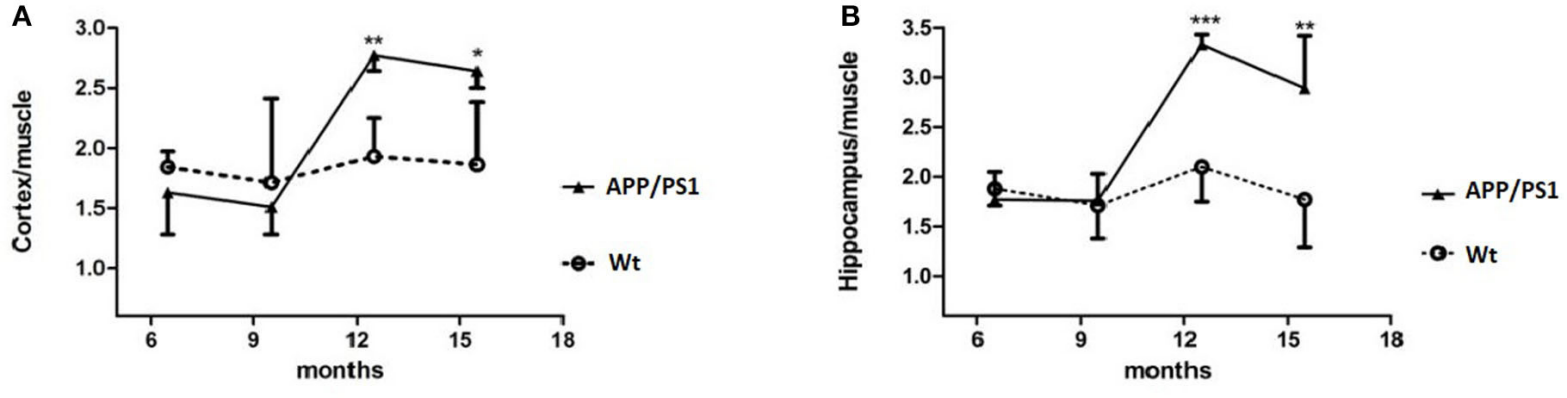
%ID/g to muscle of [^18^F]DPA714 accumulation in the cortex and hippocampus, at 6–7, 9–10, 12–13, and 15–16 months of age. ^*^p < 0.05, ^**^p < 0.01, and ^***^p < 0.001 compared with Wt age-matched mice (*T* test).

Quantitative analysis showed that there was no significant difference of [^18^F]DPA714 uptake in our defined VOIs for 6–7-months-old (cortex/muscle: 1.63 ± 0.35 vs. 1.84 ± 0.13, *P* = 0.13; hippocampus/muscle: 1.77 ± 0.28 vs. 1.88 ± 0.17, *P* = 0.24; *n* = 8) and 9–10-months-old (cortex/muscle: 1.51 ± 0.23 vs. 1.71 ± 0.70, *P* = 0.29; hippocampus/muscle: 1.76 ± 0.27 vs. 1.71 ± 0.33, *P* = 0.40; *n* = 8) APP/PS1 and Wt mice. We demonstrated the values by %ID/g and selected muscle as a reference region for signal-to-background ratios (Figure 4). There was significantly higher tracer accumulation in our defined VOIs for 12–13 and 15–16-months-old APP/PS1 mice (Figure 5). In the cortex (Figure 6A), we found that the cortex/muscle increased significantly (~40%) in APP/PS1 vs. Wt mice at 12–13 months (cortex/muscle: 2.77 ± 0.13 vs. 1.93 ± 0.32, ^**^P = 0.0014, *n* = 8) and 15–16 months (cortex/muscle: 2.64 ± 0.14 vs. 1.86 ± 0.52, ^*^P = 0.0159, *n* = 8). In the hippocampus (Figure 6B), the hippocampus/muscle were increased significantly (~60%) in APP/PS1 vs. Wt mice at 12–13 months (hippocampus/muscle: 3.33 ± 0.10 vs. 2.10 ± 0.35, ^***^P = 0.0008; *n* = 8) and 15–16 months (hippocampus/muscle: 2.89 ± 0.53 vs. 1.77 ± 0.48, ^**^P = 0.0050; *n* = 8).

### 3.3. Immunofluorescence Staining

**Figure 7:**
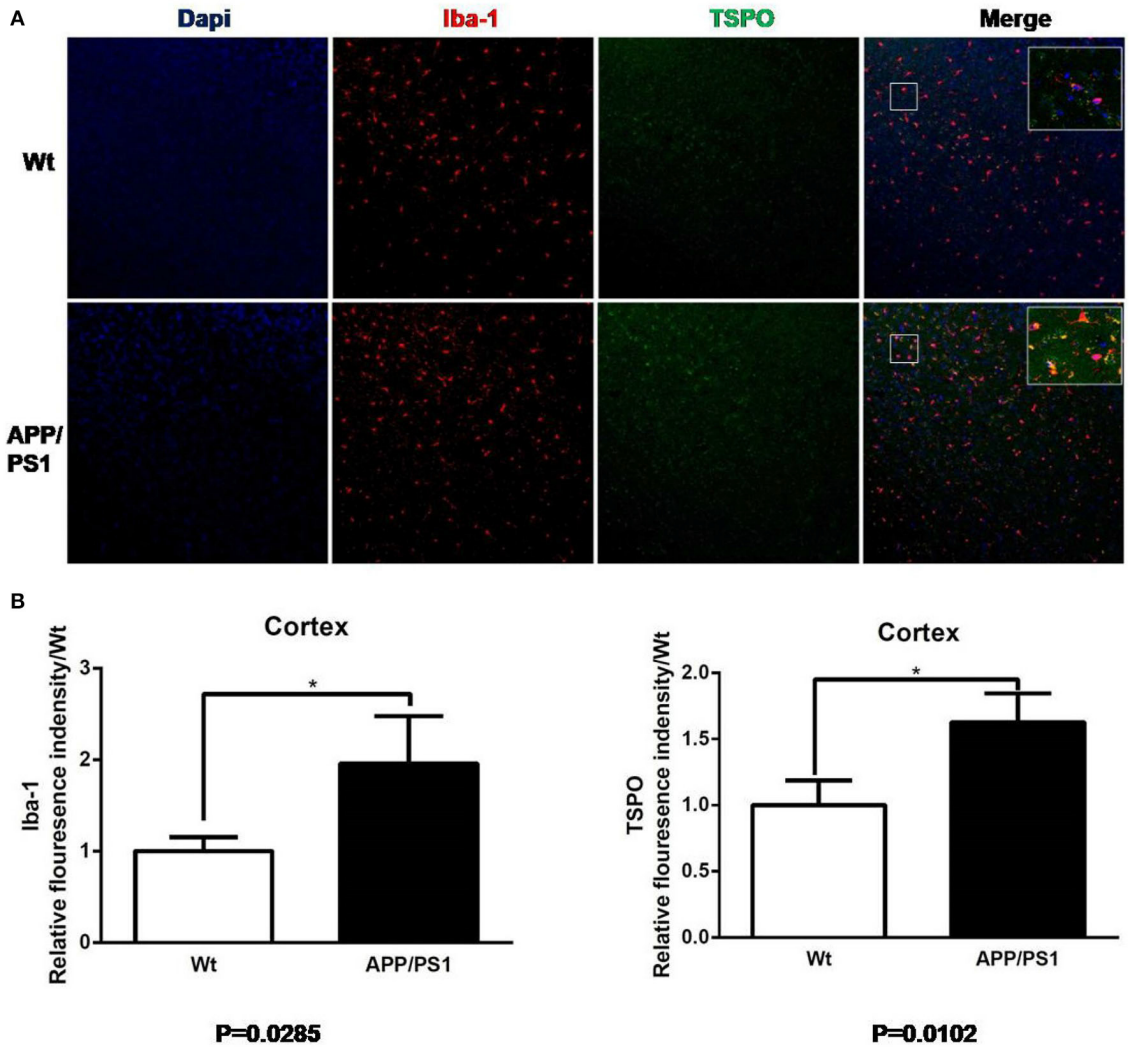
Iba-1 and TSPO staining in cortex. **(A)** Representative images from 15- to 16-months-old APP/PS1 and Wt mice, Scale bar = 50 um, magnification, ×20. **(B)** Quantitation of TSPO and Iba-1 relative fluorescence intensity in the cortex of APP/PS1 mice relative to Wt mice. *p < 0.05.

**Figure 8:**
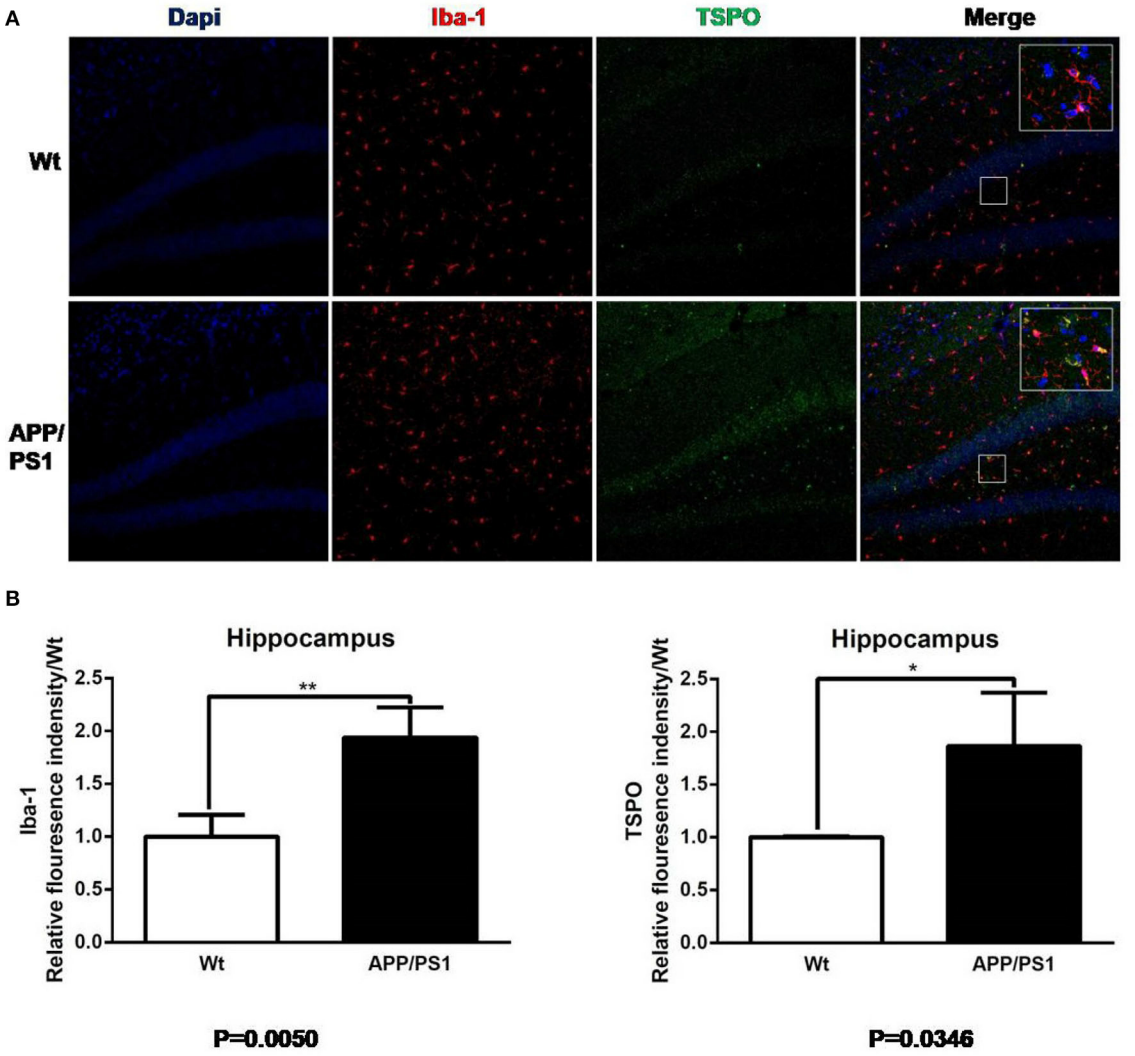
Iba-1 and TSPO staining in hippocampus. **(A)** Representative images from 15- to 16-months-old APP/PS1 and Wt mice, Scale bar = 50 um, magnification, ×20. **(B)** Quantitation of TSPO and Iba-1 relative fluorescence intensity in the hippocampus of APP/PS1 mice relative to Wt mice. *p < 0.05, **p < 0.01.

We reaped brain tissues from the 15–16-months-old mice and performed immunofluorescences staining to verify whether the [^18^F]DPA714 PET signals were associated with activated microglia and TSPO levels (Figures 7, 8). Figure 7A shows the staining figures in the cortex of Dapi, Iba-1, and TSPO and their merged figure with Wt and APP/PS1 mice. These figures illustrated that compared with Wt mice, APP/PS1 mice had much more activated microglia and a significant higher TSPO level. Figure 7B shows the relative fluorescence intensity between Wt and APP/PS1 mice with Iba-1 and TSPO. When the intensity value of Wt mice for Iba-1 formulated to 1.0, the relative fluorescence intensity of APP/PS1 mice was 1.87, and the result of *T* test was ^*^P = 0.0285 (*n* = 3 × 8). For TSPO, the relative value was 1.67 and ^*^P = 0.0102 (*n* = 3 × 8). The quantitative analysis demonstrated that there was a significant difference between Wt and APP/PS1 mice in the activated microglia and TSPO level.

**Figure 9:**
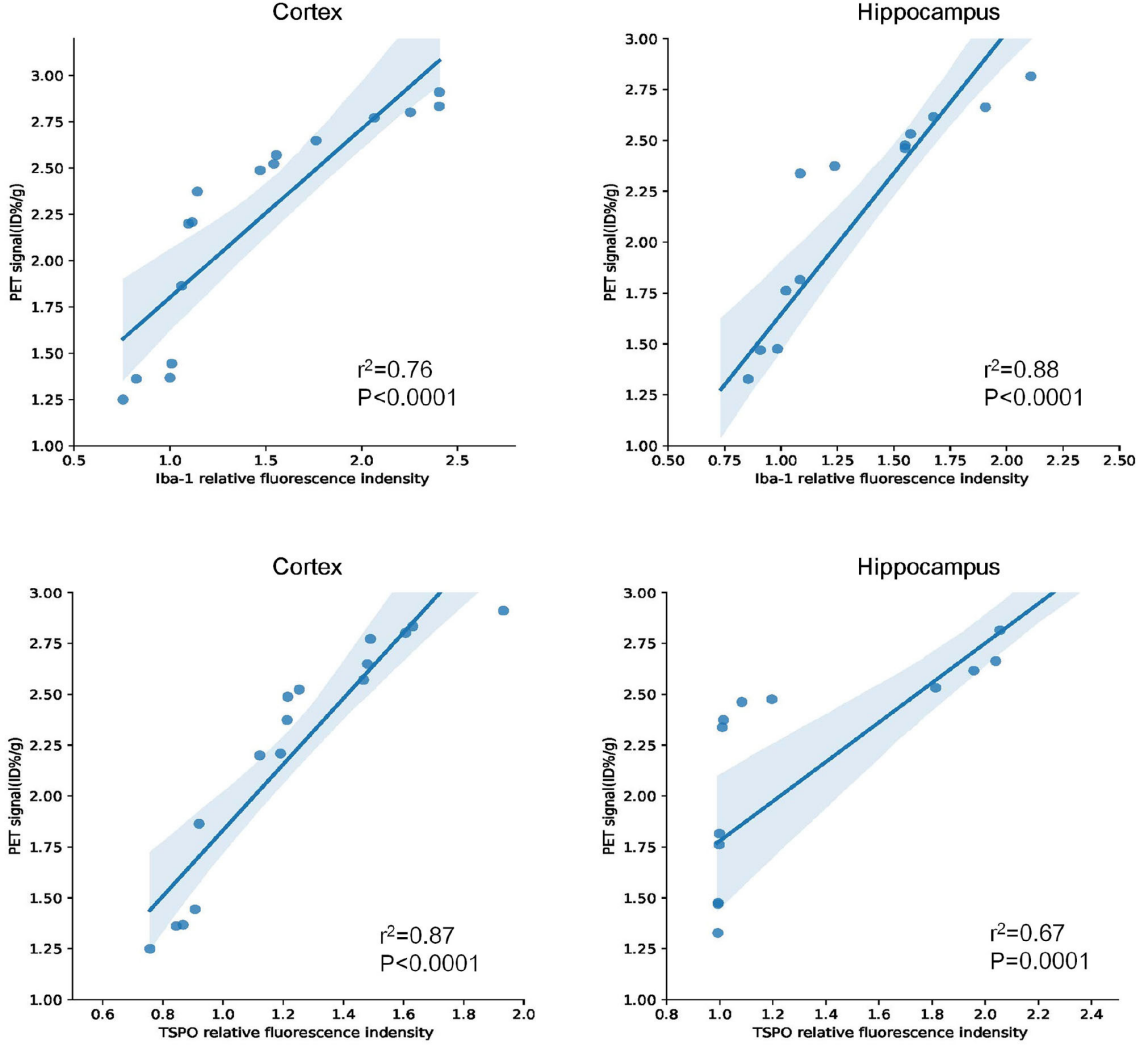
Pearson correlation analysis between Iba-1/TSPO relative fluorescence intensity and PET signal in the cortex and hippocampus for 15–16-months-old APP/PS1 and Wt mice (*n* = 8/group).

Similarly, using the same immunofluorescence staining, Figure 8 also provides the results of the staining figures in chart (A) and the relative fluorescence intensity chart in (B) for the hippocampus. In chart (B), for Iba-1, the relative fluorescence intensity of APP/PS1 mice was 1.95, and the result of the *T* test was ^**^P = 0.0050 (*n* = 3 × 8). For TSPO, the relative value was 1.81 and ^*^P = 0.0346 (*n* = 3 × 8). From the visual analysis in (A) and the quantitative analysis in (B), Figure 8 illustrates that for the hippocampus, APP/PS1 mice also had much more activated microglia and a significantly higher TSPO level than Wt mice. Additionally, in Figure 9, we conducted Pearson correlation analysis and found an obvious correlation between PET signal and Iba-1 relative fluorescence intensity in the cortex (*r*^2^ = 0.76) and hippocampus (*r*^2^ = 0.88) and also between PET signal and TSPO relative fluorescence intensity in the cortex (*r*^2^ = 0.87) and hippocampus (*r*^2^ = 0.67).

## 4. Discussion

Many studies have shown that TSPO is highly expressed in activated microglia, which makes it a potential target for neuroinflammation imaging. [^18^F]DPA714 PET has been used for various neuroinflammation models (Doorduin et al., 2009; Martín et al., 2009; Boutin et al., 2013; Lavisse et al., 2014; Wang et al., 2014). To the best of our knowledge, few tests of [^18^F]DPA714 in a well-characterized, age-related APP/PS1 mouse model of AD (Trinchese et al., 2004) have been reported. Our study used [^18^F]DPA714 PET to dynamically monitor the neuroinflammatory progression in a mouse model of AD and to ascertain the possibility of [^18^F]DPA714 as an alternative indicator for therapy response in future treatment monitoring studies. In this study, [^18^F]DPA714 PET could inspect the activation of microglia and the raised TSPO in the cortex and hippocampus of APP/PS1 mice at 12–13 and 15–16 months old. We confirmed the specificity of [^18^F]DPA714 for TSPO in the APP/PS1 mice by blocking experiments using PK11195.

AD is one of the most common neurodegenerative diseases, which is characterized by memory disorder, aphasia and dementia. Although many scholars have proposed that amyloid deposition and neurofibrillary tangles are related to the disease process of AD, the specific neuropathological changes are still poorly understood, especially for neuroinflammation. In clinical studies, it is relatively difficult to study the neuroinflammatory progression of AD patients, so, the double transgenic mouse model of APP/PS1 is often chosen to study, so as to simulate neuroinflammation (Yu et al., 2009). Poisnel et al. found that the [^18^F]-FDG uptake in the brain of APP/PS1 mice were positively correlated with the increase of age. Compared with the age-matched control mice, the uptake of [^18^F]-FDG in the cortex and hippocampus of 12-months-old APP/PS1 mice were higher (Poisnel et al., 2011). André et al. (2012) reported [^11^C]-PiB uptake in individual brain regions with Aβ deposition in the APP/PS1 mice as young as 9 months. At present, many PET tracers targeting TSPO, such as [^11^C]PK11195 (Venneti et al., 2008) and [^18^F]PBR06 (James et al., 2015) have been utilized in AD mouse models. In Venneti et al. (2008) study, [^11^C]PK11195 PET imaging showed that the concentration of tracers in the brain of APP/PS1 mice increased progressively, which related to the histopathological abundance of activated microglia detected by Iba-1 staining. Another study (James et al., 2015) reported that [^18^F]PBR06, another potential imaging agent for neuroinflammation, gained higher uptake in the cortex and hippocampus of 15–16-months-old APP^*L*/*S*^ mice than Wt, and PET results correlated well with immunostaining. Our study revealed that Iba-1, a marker of activated microglia, is detected in the cortex and hippocampus by immunohistochemistry staining. Additionally, we found that TSPO is highly increased in the cortex and hippocampus of 15–16-months-old APP/PS1 mice, which is co-localized with Iba-1, indicating that TSPO is highly expressed in activated microglia. These results suggest that activated microglia are involved in the disease development of neuroinflammation in AD mice, and elevated TSPO expression can be detected by PET imaging. Thus, activated microglia may be used as an effective target for the treatment of neuroinflammatory damage of AD in the future.

A non-invasive PET imaging with [^18^F]DPA714 for microglia has been used in clinical trials. Arlicot et al. (2012) showed that [^18^F]DPA714 is a promising PET radioligand with excellent *in vivo* bio-distribution and acceptable effective dose estimation in healthy humans. Although the initial clinical studies had shown that [^18^F]DPA714 may not be suitable for early diagnosis of AD patients (Golla et al., 2015), the potential role of [^18^F]DPA714 in monitoring TSPO for AD requires further research. Lorraine et al. (2016) found that microglia are activated in the early stages of AD and play a protective role in the disease. Subsequent work will involve using [^18^F]DPA714 to monitor the anti-inflammatory treatment in an AD mouse model, which may be helpful in finding a better treatment method for AD.

## 5. Conclusion

In this study we optimized the radioactive labeling procedure of [^18^F]DPA714 to improve its radiochemical yield and performed a longitudinal PET study that demonstrated increased inflammation in the brains of APP/PS1 mice compared to Wt mice. Results showed significantly higher signals in our defined VOIs of 12–13 and 15–16 months old APP/PS1 than Wt mice, with levels of TSPO and activated microglia, and no significant differences in [^18^F]DPA714 PET signals between younger APP/PS1 and Wt mice. This finding might lead to the selection of mouse age when using [^18^F]DPA714 to study the anti-inflammatory treatment of a APP/PS1 mouse model in the future. Our results suggest that [^18^F]DPA714, a molecular probe targeting TSPO, has great potential in monitoring microglia activation and neuroinflammation and discovering the best time point of anti-inflammatory therapy for Alzheimer's disease.

## Data Availability Statement

The raw data supporting the conclusions of this article will be made available by the authors, without undue reservation.

## Ethics Statement

The animal study was reviewed and approved by National Institute of Health Guide for the Care and Use of Laboratory Animals.

## Author Contributions

WH together with DP and YW performed the *in vivo* and immunofluorescence experiments, and JZ, MY, and WB analyzed and interpreted the data and drafted the manuscript and the figures. WH and DP synthesized the radiotracers. CZ, YG, and FH provided the specific input on the experimental design, data acquisition, and analysis and contributed to manuscript writing. JZ and MY conceived and designed the study and critically revised the manuscript. All authors have seen and agree with the content of the manuscript.

## Conflict of Interest

The authors declare that the research was conducted in the absence of any commercial or financial relationships that could be construed as a potential conflict of interest.
